# Timing of Palliative Care, End-of-Life Quality Indicators, and Health Resource Utilization

**DOI:** 10.1001/jamanetworkopen.2024.40977

**Published:** 2024-10-28

**Authors:** Sarah J. Mah, Daniel M. Carter Ramirez, Kara Schnarr, Lua R. Eiriksson, Anastasia Gayowsky, Hsien Seow

**Affiliations:** 1 Department of Obstetrics and Gynecology, Division of Gynecologic Oncology, McMaster University, Hamilton, Ontario, Canada; 2 Department of Family Medicine, Division of Palliative Care, McMaster University, Hamilton, Ontario, Canada; 3 Department of Oncology, Division of Radiation Oncology, McMaster University, Hamilton, Ontario, Canada; 4 ICES McMaster, Hamilton, Ontario, Canada; 5 Department of Oncology, McMaster University, Hamilton, Ontario, Canada

## Abstract

**Importance:**

Despite research supporting the benefits of early palliative care, timely initiation by gynecologic oncology patients is reportedly low, which may limit the effectiveness of palliative care.

**Objective:**

To investigate the association of the timing of palliative care initiation with the aggressiveness of end-of-life care using established quality indicators among patients with ovarian cancer.

**Design, Setting, and Participants:**

This population-based retrospective cohort study of ovarian cancer decedents used linked administrative health care data to identify palliative care provision across all health care sectors and health care professionals (specialist and nonspecialist) and end-of-life quality indicators in Ontario, Canada, from 2006 to 2018. Data analyses were performed July 12, 2024.

**Main Outcomes and Measures:**

The primary outcome was the associations between the timing of palliative care and end-of-life quality indicators, including emergency department use, hospital or intensive care unit admission in the last 30 days of life, chemotherapy in last 14 days of life, death in the hospital, and a composite measure of aggressive care. Late palliative care was defined as 3 months or less prior to death.

**Results:**

There were 8297 ovarian cancer decedents. Their mean (SD) age at death was 69.6 (13.1) years, and their mean (SD) oncologic survival was 2.8 (3.9) years. Among 3958 patients with known cancer stage, 3495 (88.3%) presented with stage III or IV disease. One-third of patients (2667 [32.1%]) received late palliative care in the final 3 months of life. Results of multivariable regression analysis indicated that any palliative care initiated earlier than 3 months before death was associated with lower rates of aggressive end-of-life care (odds ratio [OR], 0.47 [95% CI, 0.37-0.60]), death in hospital (OR, 0.54 [95% CI, 0.45-0.65]), and intensive care unit admission (OR, 0.46 [95% CI, 0.27-0.76]). Specialist palliative consultation from 3 months up to 6 monts before death was associated with decreased likelihood of late chemotherapy (OR, 0.46 [95% CI, 0.24-0.88]).

**Conclusions:**

Findings from this cohort study suggested that early palliative care may be associated with less-aggressive end-of-life care than late palliative care. Implementation strategies for early palliative care initiation are needed to optimize care quality and health resource utilization at the end of life.

## Key Points

**Question:**

When should palliative care be initiated to reduce the likelihood of aggressive end-of-life care for patients with ovarian cancer?

**Findings:**

In this cohort study of 8297 individuals with ovarian cancer decedents, initiating palliative care earlier than 3 months before death was associated with lower rates of death in the hospital, late chemotherapy, intensive care unit admission, and aggressive care at the end-of-life.

**Meaning:**

Findings from this cohort study suggest that strategies to increase uptake of early palliative care more than 3 months before death may decrease the aggressiveness of end-of-life care.

## Teaser

This cohort study investigates whether the timing of palliative care initiation is associated with the aggressiveness of end-of-life care among patients with ovarian cancer.

## Introduction

The American Society of Clinical Oncology recommends that all patients with advanced cancer, including metastatic or life-limiting disease with a prognosis of 6 to 24 months, receive early, dedicated palliative care (PC) within 8 weeks of diagnosis in parallel with oncologic treatment.^1^ Benefits of PC in patients with cancer include improved patient and caregiver quality of life (QOL) and symptom management, prolonged survival, and enhanced quality of end-of-life (EOL) care.^1,2,3^ With increasing recognition that futile and overly aggressive care at the EOL is costly to patients and the health care system,^3,4^ studies in the general cancer population have reported that early specialist PC referral is associated with less-aggressive EOL care, including emergency department (ED) visits, hospital and intensive care unit (ICU) admissions, and inpatient death.^5,6^

The optimal timing of early PC initiation that balances patient care quality with finite PC resources remains unknown for patients with gynecologic cancers^3,7^ and is particularly relevant given that they may experience a greater burden of physical symptoms earlier in the disease course and more progressive anxiety and depression, and report decreased QOL compared with patients with other solid tumors.^8,9,10,11^ A previous population-based study of patients with gynecologic cancer reported that despite greater access to PC over time, there was no corresponding decrease in the intensity of EOL care.^12^ Further, a retrospective cohort study—in which nearly 40% of ovarian cancer decedents received PC consultation only in the final 30 days of life—did not find an association between PC consultation and EOL quality indicators, including late chemotherapy administration, death in the hospital, or hospice referral.^13^ These studies suggest that the method and timing of PC delivery are key to its effectiveness. The objective of the present study was to examine the association of timing of palliative care initiation, including care by specialist and nonspecialist primary care clinicians, with the aggressiveness of EOL care in gynecologic oncology.

## Methods

### Population and Study Design

This retrospective cohort study used linked administrative data from ICES (formerly the Institute for Clinical Evaluative Sciences) to identify patients in the province of Ontario, Canada, who died of ovarian cancer between January 1, 2006, and December 31, 2018, using the *International Statistical Classification of Diseases, Tenth Revision* (*ICD-10*).^14^ We excluded individuals who were 18 years or younger at time of death or with an invalid provincial health card number. This study was approved by the Hamilton Integrated Research Ethics Board, which also granted a waiver for the need to obtain participant informed consent given the deidentified nature of the aggregate data. This manuscript was prepared in adherence to the Strengthening the Reporting of Observational Studies in Epidemiology (STROBE) reporting guideline for cohort studies.^15^

### Data Sources and Definitions

Encrypted provincial health care numbers were used to link patient data to the following administrative health databases: Ontario Cancer Registry (cancer diagnosis and cause of death), Canadian Institute for Health Information Discharge Abstract Database (diagnostic and procedure information for acute care hospitalizations), Ontario Health Insurance Plan database (physician billing claims and payments), National Ambulatory Care Reporting System for ED visits, Activity Level Reporting (chemotherapy use), Home Care database, Client Agency Program Enrolment (to identify patients with a primary care clinician), and Statistics Canada (sociodemographic variables).^16,17^ A modified Deyo-Charlson Comorbidity Index (DCCI) score was calculated using *ICD-10* codes from hospital admissions from 36 months prior to death to 1 month prior to death,^18,19^ excluding the gynecologic cancer attributed to cause of death.

Palliative care provision was determined using previously validated administrative codes.^20^ Physician billing claims across all specialties were captured and coded by sector, with institutional PC provided in acute care hospitals, complex continuing care facilities, and long-term care homes, and community PC delivered in ambulatory clinics, as designated EOL home care, or in hospice or PC units. These claims included physician billings for PC support, case management, telephone consultations, and home visits, with unique codes for specialist and nonspecialist physicians (including oncologists and primary care physicians). Specialist PC initiation was defined as the first billing of an inpatient or outpatient special PC consult. We also captured nonspecialist palliative home care provided by nurses, personal support workers, and other allied health. We defined late PC as services received less than 3 months prior to death, based on the American Society of Clinical Oncology recommendation,^1^ surveys of patients with advanced cancer, and prior international Delphi consensus research on optimal PC timing in which there was 70% or higher agreement that referral to PC with an estimated prognosis of less than 3 months was too late.^21,22,23^ We also examined decedents who did not receive any PC services as a separate comparator group.

### Health Service EOL Quality Indicators

Our EOL quality indicators were previously described in this cohort^12^ and were derived from a systematic review of population-based EOL quality care indicators by Henson et al,^24^ including indicators endorsed by the National Quality Forum.^25^ We defined EOL as the final 30 days of life and included the following indicators that were measurable with our administrative data: (1) at least 1 new hospitalization in the final 30 days, (2) at least 1 ED visit in the final 14 or 30 days, (3) at least 1 new ICU admission in the final 30 days, (4) at least 1 physician house call in the final 14 days, and (5) chemotherapy in the final 14 days (late chemotherapy). We also determined the proportion of decedents who received any nonphysician PC home care service in the final 30 days and the proportion who died in the hospital.

Per prior research,^4,12,26^ these individual quality indicators were combined to form 2 aggregate, nonmutually exclusive indicators: aggressive and supportive care. Aggressive care was defined as 1 or more of the following: (1) 2 or more ED visits in the final 30 days, (2) 2 or more new hospital admissions within the final 30 days, or (3) 1 or more new ICU admission in the final 30 days. Supportive care was defined as 1 or more of the following: (1) at least 1 physician house call in the final 14 days or (2) at least 1 PC home care service in the final 30 days. Thus, patients could receive aggressive or supportive care or both at the EOL.

### Statistical Analysis

Descriptive statistics were used to characterize the population at the time of death. Multivariable logistic regression models were generated to assess the association of the timing of PC initiation with the likelihood of receiving aggressive or supportive EOL care and with individual EOL quality indicators. We excluded patients from these models with oncologic survival less than 3 months as these patients would by default be unable to receive PC earlier than our late definition. Separate models were generated to examine the association of PC with different health care professionals (specialist vs nonspecialist PC), as well as for subcohorts with known and missing cancer stage data. Covariates included in the adjusted models were timing of PC initiation using these categories, age at death, oncologic survival measured as the time from gynecologic cancer diagnosis to death, DCCI score (range, 0-17, with comorbidities present defined as a score of 1 or higher), cancer stage at diagnosis, whether the patient had a primary care clinician, rurality (community population <10 000) and neighborhood income quintile, and calendar year of death.

Statistical analyses were performed July 12, 2024, using SAS, versions 9.3 and 9.4 (SAS Institute Inc), R, version 3.0.1 (R Project for Statistical Computing), and Microsoft Excel 2021. Python NumPy (Python Software Foundation) was used for data visualization.^16^Statistical significance was defined as a 95% CI excluding 1.

## Results

### Demographics

There were 8297 included ovarian cancer decedents (Table 1). Their mean (SD) age at death was 69.6 (13.1) years. The mean (SD) length of oncologic survival was 2.8 (3.9) years. Among 3958 patients (47.7%) for whom cancer stage data were available, 2366 (59.8%) presented with stage III disease, and 1129 (28.5%) presented with stage IV disease. Of 5759 decedents with available DCCI data, 3809 (66.1%) had a score of 1 or higher. The characteristics of 4339 decedents for whom cancer stage data were unavailable are summarized in eTable 1 in Supplement 1.

**Table 1.** Characteristics of the Ovarian Cancer Decedent Cohort

Characteristic	Patients, No. (%) (n = 8297)
Age group at death, y	
18-39	115 (1.4)
40-49	449 (5.4)
50-59	1340 (16.2)
60-69	2008 (24.2)
70-79	2303 (27.8)
≥80	2082 (25.1)
Cancer stage at diagnosis	
I	223 (2.7)
II	240 (2.9)
III	2366 (28.5)
IV	1129 (13.6)
Unknown or missing data	4339 (52.3)
Length of survival from gynecologic cancer diagnosis	
<30 d	733 (8.8)
≥1 up to 3 mo	843 (10.2)
≥3 up to 6 mo	568 (6.9)
≥6 up to 12 mo	864 (10.4)
≥12 mo up to 5 y	3968 (47.8)
≥5 y	1321 (15.9)
Timing of palliative care initiation prior to death	
No palliative care	327 (3.9)
<30 d	1304 (15.7)
≥1 up to 3 mo	1363 (16.4)
≥3 up to 6 mo	947 (11.4)
≥6 up to 12 mo	1175 (14.2)
≥12 mo	3181 (38.3)
Score on the Deyo-Charlson Comorbidity Index (from 36 mo to 1 mo before death)^a^	
0	1950 (23.5)
≥1	3809 (45.9)
Unknown or missing data	2538 (30.6)
Patient had a primary care clinician in CAPE as of diagnosis date	
Yes	5939 (71.6)
No	2358 (28.4)
Rural status and income quintile	
Rural	1131 (13.6)
Urban income quintile 1 (lowest)	1484 (17.9)
Urban income quintile 2	1459 (17.6)
Urban income quintile 3	1357 (16.4)
Urban income quintile 4	1415 (17.1)
Urban income quintile 5 (highest)	1433 (17.3)
Unknown or missing data	18 (0.2)

Abbreviations: CAPE, Client Agency Program Enrolment;

^a^ Scores may range from 0 to 17, with comorbidities present defined as a score of 1 or higher.

### Timing of Palliative Care Initiation

Of 8297 decedents, 7970 (96.1%) received PC that was initiated a median (IQR) of 238 (55-633) days before death. Of decedents who accessed any PC, it was initiated late for 2667 patients (32.1%): 1304 (15.7%) initiated PC in the final 30 days of life, and 1363 (16.4%) from 1 up to 3 months before death (eTable 2 in Supplement 1). In addition, 947 patients (11.4%) initiated PC from 3 months up to 6 months before death, 1175 patients (14.2%) from 6 months up to 12 months before death, and 3181 patients (38.3%) 12 or more months before death. The timing of PC initiation was similar among patients diagnosed with early (stage I-II) vs advanced (stage III-IV) cancers (eTable 2 in Supplement 1).

### Timing of Palliative Care and EOL Quality Indicators

As depicted in Table 2 and the Figure, decedents who initiated any PC late had higher rates of individual aggressive EOL care quality indicators compared with decedents who received earlier PC. This included higher rates compared with earlier PC for ED visits in the final 14 days of life (43.1% with late PC vs 25.2%-30.7%), new hospitalization in the final 30 days of life (68.3% vs 42.0%-50.2%), new ICU admission in the final 30 days of life (8.9% vs 2.8%-3.7%), late chemotherapy (7.8% vs 4.2%-4.6%), and death in the hospital (56.3% vs 36.7%-38.3%). The overall rate of aggressive EOL care was 29.7% with late PC vs 15.8% to 18.2% with earlier PC.

**Table 2.** Frequency of End-Of-Life Quality Indicators by Timing of Palliative Care Initiation

End-of-life quality indicator with inclusion definition	Timing of palliative care initiation before death, No. (%) of patients				
	No palliative care	<3 mo	≥3 up to 6 mo	≥6 up to 12 mo	≥12 mo
No. in full study population	327	2667	947	1175	3181
Death in acute care hospital bed	194 (59.3)	1502 (56.3)	353 (37.3)	450 (38.3)	1168 (36.7)
Total days in hospital in last 30 d of life					
Mean (SD)	8.9 (9.9)	12.5 (10.3)	8.5 (10.6)	8.1 (9.7)	7.7 (9.7)
Median (IQR)	5 (0-14)	11 (3-21)	3 (0-15)	4 (0-14)	3 (0-13)
Any palliative care service in last year of life	0	2667 (100)	947 (100)	1175 (100)	3140 (98.7)
No. of patients not in hospital for all of last 30 d of life	301	2356	848	1089	2956
New hospitalization in last 30 d of life	189 (62.8)	1610 (68.3)	356 (42.0)	547 (50.2)	1397 (47.3)
New ICU admission in last 30 d of life	58 (19.3)	209 (8.9)	24 (2.8)	40 (3.7)	97 (3.3)
Any palliative care service in last 30 d of life	0	2306 (97.9)	810 (95.5)	1043 (95.8)	2842 (96.1)
No. of patients not in hospital for all of last 14 d of life	251	1902	759	989	2661
Emergency department visit in last 14 d of life	131 (52.2)	819 (43.1)	191 (25.2)	304 (30.7)	755 (28.4)
Chemotherapy use in last 14 d of life	38 (15.1)	149 (7.8)	35 (4.6)	44 (4.5)	111 (4.2)
Physician house call in last 14 d of life	0	394 (20.7)	263 (34.7)	335 (33.9)	1022 (38.4)
No. of patients not in hospital for all of last 30 d of life	301	2356	848	1089	2956
Aggressive care	106 (35.2)	699 (29.7)	134 (15.8)	198 (18.2)	508 (17.2)
At least 2 ED visits in last 30 d of life	57 (18.9)	484 (20.5)	99 (11.7)	149 (13.7)	375 (12.7)
At least 2 new hospitalizations within last 30 d of life	27 (9.0)	298 (12.7)	49 (5.8)	85 (7.8)	199 (6.7)
New ICU admission in last 30 d of life	68 (22.6)	202 (8.6)	23 (2.7)	32 (2.9)	101 (3.4)
Supportive care	0	999 (42.4)	567 (66.9)	741 (68.0)	2094 (70.8)
Physician house call in last 14 d of life	0	398 (16.9)	264 (31.1)	336 (30.9)	1025 (34.7)
Any palliative care home care service in last 30 d of life	0	934 (39.6)	534 (63.0)	702 (64.5)	1985 (67.2)

Abbreviation: ED, emergency department; ICU, intensive care unit.

**Figure.**
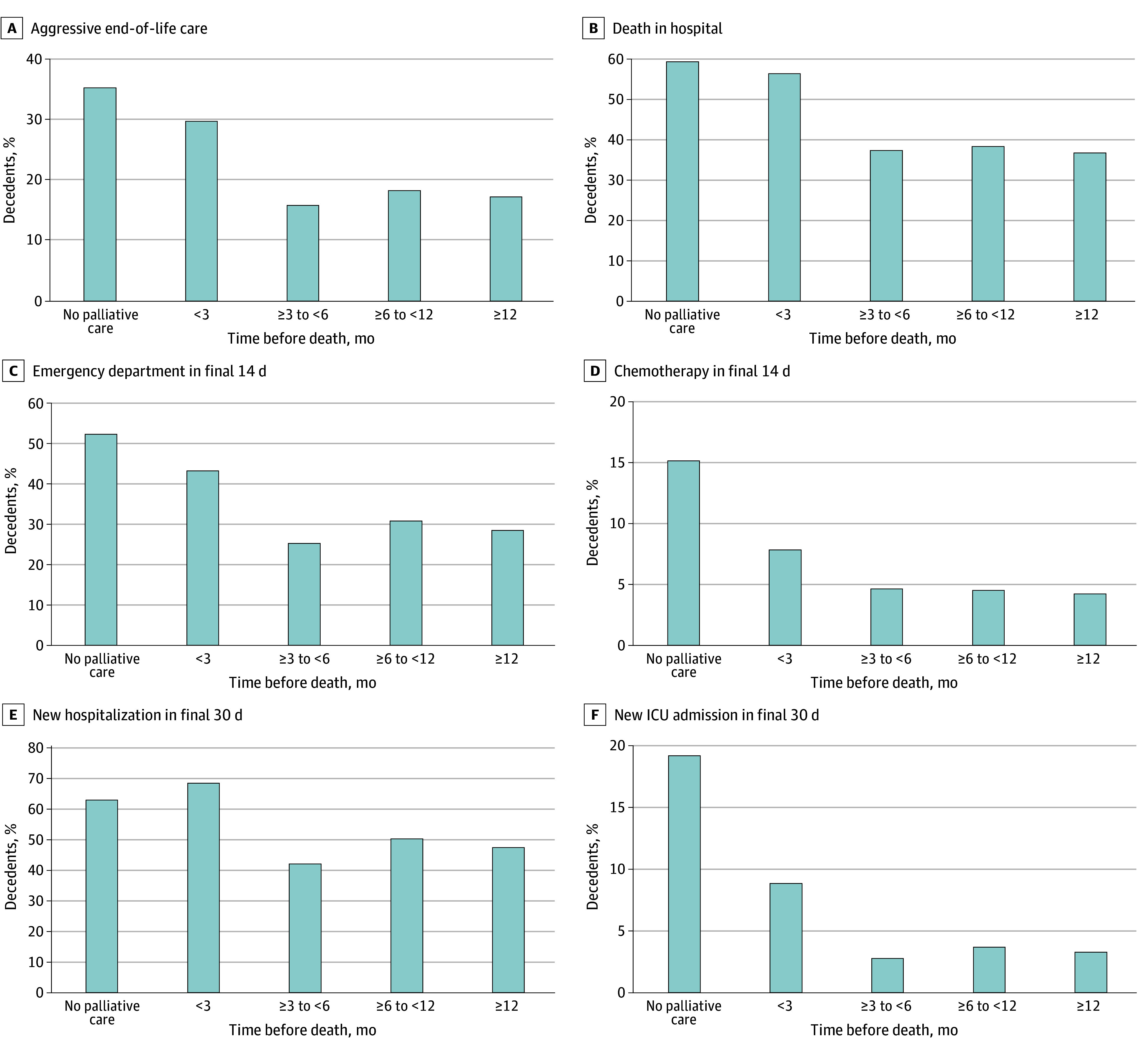
Proportion of Ovarian Cancer Decedents Experiencing Each End-of-Life Quality Indicator by Timing of Palliative Care Initiation ICU indicates intensive care unit.

The results of our multivariable analysis (Table 3) indicated that compared with decedents with late PC, decedents with PC from 3 months up to 6 months before death were significantly less likely to receive aggressive EOL care (odds ratio [OR], 0.47 [95% CI, 0.37-0.60]), be admitted to ICU in their final 30 days (OR, 0.46 [95% CI, 0.27-0.76]), or die in the hospital (OR, 0.54 [95% CI, 0.45-0.65]). They were also significantly more likely to access supportive care at EOL (OR, 1.98 [95% CI, 1.63-2.42]). The presence of the associations persisted when comparing individuals who initiated PC late and patients who received PC even earlier, from 6 months up to 12 months or 12 months or longer before death (Table 3) and were also observed for patients with missing stage data, with the exception of ICU admission (eTable 3 in Supplement 1). There was no difference in the likelihood of receiving late chemotherapy between patients who received late vs earlier PC overall.

**Table 3.** Multivariable Logistic Regression Analysis for Aggregate and Individual End-Of-Life Quality Indicators, Including All Palliative Care

Indicator	OR (95% CI)				
	Aggressive care	Supportive care	New ICU admission (last 30 d)	Chemotherapy use (last 14 d)	Death in hospital
Age group at death, y					
18-39	2.26 (1.45-3.53)	1.64 (0.96-2.79)	2.42 (1.16-5.03)	0.65 (0.20-2.15)	1.76 (1.17-2.65)
40-49	1.33 (1.00-1.76)	1.14 (0.86-1.50)	1.57 (0.95-2.57)	1.25 (0.75-2.10)	1.47 (1.17-1.85)
50-59	1 [Reference]	1 [Reference]	1 [Reference]	1 [Reference]	1 [Reference]
60-69	0.90 (0.74-1.09)	0.88 (0.74-1.05)	0.82 (0.56-1.18)	1.12 (0.79-1.58)	0.88 (0.76-1.03)
70-79	0.73 (0.61-0.89)	0.72 (0.61-0.85)	0.64 (0.44-0.95)	0.69 (0.47-1.00)	0.80 (0.69-0.93)
≥80	0.52 (0.42-0.65)	0.62 (0.52-0.74)	0.39 (0.24-0.63)	0.36 (0.22-0.57)	0.56 (0.47-0.66)
Oncologic survival from cancer diagnosis to death					
≥3 up to 6 mo	0.87 (0.64-1.19)	1.16 (0.88-1.52)	1.03 (0.54-1.98)	1.37 (0.77-2.45)	1.20 (0.93-1.53)
≥6 up to 12 mo	1 [Reference]	1 [Reference]	1 [Reference]	1 [Reference]	1 [Reference]
≥1 up to 5 y	0.72 (0.58-0.90)	1.33 (1.09-1.61)	0.94 (0.59-1.50)	0.88 (0.57-1.35)	0.84 (0.70-1.01)
≥5 y	0.65 (0.50-0.86)	1.30 (1.03-1.64)	1.19 (0.69-2.03)	0.68 (0.40-1.17)	0.79 (0.64-0.97)
Deyo-Charlson Comorbidity Index score^a^					
0 or Missing	1 [Reference]	1 [Reference]	1 [Reference]	1 [Reference]	1.00
≥1	0.91 (0.80-1.04)	0.94 (0.84-1.05)	0.98 (0.75-1.28)	0.90 (0.70-1.16)	0.96 (0.87-1.06)
Cancer stage					
I	1.34 (0.95-1.90)	1.00 (0.72-1.39)	0.94 (0.47-1.90)	0.73 (0.31-1.71)	1.01 (0.75-1.36)
II	0.94 (0.64-1.37)	0.87 (0.64-1.19)	1.35 (0.72-2.54)	1.57 (0.85-2.89)	0.91 (0.68-1.22)
III	1 [Reference]	1 [Reference]	1 [Reference]	1 [Reference]	1 [Reference]
IV	1.01 (0.82-1.25)	1.10 (0.92-1.32)	0.72 (0.45-1.14)	1.15 (0.78-1.71)	0.92 (0.78-1.09)
Missing	0.99 (0.83-1.17)	0.97 (0.84-1.12)	0.86 (0.61-1.22)	0.88 (0.63-1.24)	1.04 (0.91-1.19)
Rural status and income quintile					
Rural	1.79 (1.43-2.25)	0.78 (0.63-0.95)	0.65 (0.39-1.08)	1.35 (0.85-2.15)	1.66 (1.39-1.98)
Urban 1 (lowest)	1.42 (1.14-1.77)	0.60 (0.49-0.72)	1.03 (0.67-1.57)	1.35 (0.87-2.08)	1.19 (1.01-1.41)
Urban 2	1.12 (0.89-1.40)	0.65 (0.54-0.79)	0.86 (0.55-1.33)	1.03 (0.65-1.63)	1.02 (0.86-1.21)
Urban 3	1.13 (0.90-1.42)	0.74 (0.61-0.89)	0.97 (0.64-1.49)	1.42 (0.92-2.18)	1.16 (0.97-1.37)
Urban 4	1.05 (0.84-1.32)	0.79 (0.66-0.96)	0.92 (0.60-1.41)	1.25 (0.81-1.93)	1.10 (0.93-1.30)
Urban 5 (Highest)	1 [Reference]	1 [Reference]	1 [Reference]	1 [Reference]	1 [Reference]
Timing of palliative care initiation prior to death					
No palliative care	1.13 (0.77-1.65)	NA	4.88 (2.90-8.19)	2.59 (1.46-4.61)	0.92 (0.66-1.27)
<3 mo	1 [Reference]	1 [Reference]	1 [Reference]	1 [Reference]	1 [Reference]
≥3 up to 6 mo	0.47 (0.37-0.60)	1.98 (1.63-2.42)	0.46 (0.27-0.76)	0.78 (0.49-1.22)	0.54 (0.45-0.65)
≥6 up to 12 mo	0.51 (0.41-0.64)	2.07 (1.70-2.51)	0.46 (0.29-0.75)	0.78 (0.50-1.22)	0.63 (0.52-0.75)
≥12 mo	0.53 (0.44-0.65)	2.00 (1.69-2.37)	0.47 (0.32-0.69)	0.81 (0.55-1.20)	0.64 (0.55-0.75)
Primary care clinician					
No	1.03 (0.88-1.21)	0.86 (0.75-0.99)	1.07 (0.78-1.47)	0.96 (0.71-1.32)	1.06 (0.93-1.20)
Yes	1 [Reference]	1 [Reference]	1 [Reference]	1 [Reference]	1 [Reference]
Year of death					
2006	1 [Reference]	1 [Reference]	1 [Reference]	1 [Reference]	1 [Reference]
2007	0.93 (0.66-1.32)	1.04 (0.77-1.39)	1.15 (0.55-2.40)	0.84 (0.47-1.51)	0.79 (0.61-1.02)
2008	1.01 (0.71-1.44)	0.89 (0.66-1.19)	1.32 (0.62-2.82)	0.83 (0.46-1.52)	1.15 (0.89-1.50)
2009	0.88 (0.61-1.25)	1.00 (0.74-1.34)	1.48 (0.69-3.15)	0.51 (0.26-1.02)	0.85 (0.66-1.11)
2010	1.07 (0.74-1.53)	0.95 (0.71-1.28)	1.50 (0.68-3.31)	0.64 (0.33-1.23)	0.75 (0.58-0.99)
2011	1.16 (0.81-1.66)	1.21 (0.89-1.64)	1.61 (0.73-3.53)	0.72 (0.38-1.37)	0.81 (0.62-1.06)
2012	1.24 (0.86-1.78)	1.19 (0.87-1.63)	2.55 (1.21-5.38)	0.54 (0.27-1.09)	0.78 (0.59-1.03)
2013	1.21 (0.84-1.74)	1.00 (0.74-1.36)	2.79 (1.33-5.85)	0.54 (0.27-1.09)	0.85 (0.64-1.12)
2014	1.22 (0.84-1.75)	1.02 (0.75-1.39)	2.35 (1.10-5.04)	0.52 (0.26-1.04)	0.82 (0.62-1.08)
2015	1.22 (0.85-1.74)	1.26 (0.93-1.71)	2.68 (1.27-5.65)	0.57 (0.29-1.11)	0.77 (0.59-1.02)
2016	1.30 (0.91-1.85)	1.19 (0.88-1.62)	1.54 (0.68-3.49)	0.72 (0.38-1.37)	0.67 (0.51-0.89)
2017	1.36 (0.96-1.94)	1.29 (0.95-1.74)	2.56 (1.20-5.44)	0.60 (0.31-1.16)	0.69 (0.52-0.91)
2018	1.47 (1.04-2.09)	1.22 (0.90-1.66)	2.85 (1.36-5.97)	0.78 (0.41-1.47)	0.82 (0.62-1.07)

Abbreviations: ICU, intensive care unit; NA, not applicable; OR, odds ratio.

^a^ Scores may range from 0 to 17, with comorbidities present defined as a score of 1 or higher.

Patients who did not receive any PC were significantly more likely to receive late chemotherapy compared with those with late PC (OR, 2.59 [95% CI, 1.46-4.61]) and to be admitted to the ICU in their final 30 days (OR, 4.88 [95% CI, 2.90-8.19]). However, there was no difference in their likelihood of dying in the hospital (OR, 0.92 [95% CI, 0.66-1.27]) or of receiving aggressive EOL care (OR, 1.13 [95% CI, 0.77-1.65]).

### Other Associations With EOL Quality Indicators

When examining covariates in the multivariable analysis, patients 40 to 49 years of age were more likely to receive aggressive care (OR, 1.33 [95% CI, 1.00-1.76]) and die in the hospital (OR, 1.47 [95% CI, 1.17-1.85]), while patients 70 to 79 years of age were significantly less likely to receive either aggressive care (OR, 0.73 [95% CI, 0.61-0.89]) or supportive care (OR, 0.72 [95% CI, 0.61-0.85]) as were patients 80 years or older (OR, 0.52 [95% CI, 0.42-0.65] for aggressive care; OR, 0.62 [95% CI, 0.52-0.74] for supportive care) (Table 3). An oncologic survival of at least 1 year to 5 years was associated with significantly lower likelihood of aggressive care (OR, 0.72 [95% CI, 0.58-0.90]) and significantly higher likelihood of supportive care (OR, 1.33 [95% CI, 1.09-1.61]). Patients living in rural communities were significantly more likely to receive aggressive EOL care (OR, 1.79 [95% CI, 1.43-2.25]) and die in the hospital (OR, 1.66 [95% CI, 1.39-1.98]), as were patients in the lowest urban income quintile (OR, 1.42 [95% CI, 1.14-1.77] for aggressive care; OR, 1.19 [95% CI, 1.01-1.41] for dying in the hospital), and less likely to receive supportive EOL care (OR, 0.78 [95% CI, 0.63-0.95] for rural; OR, 0.60 [95% CI, 0.49-0.72] for lowest urban income quintile), compared with the highest income urban quintile. From 2012 to 2018 compared with 2006, decedents were approximately 2.5 times more likely to be admitted to ICU in the final 30 days, with no other definite trends in quality indicators over time. There were no associations between DCCI score or cancer stage and EOL outcomes. In our multivariable regression analysis including only decedents with known cancer stage, there was still no association between stage and any EOL outcome (eTable 4 in Supplement 1).

### Associations of Specialist and Nonspecialist Palliative Care Provision

Only 4298 patients (51.8%) received a specialist PC consultation, with similar rates of specialist PC between patients diagnosed with early and advanced stage cancers (eTable 2 in Supplement 1). The presence of all associations between timing of PC and aggressiveness of EOL care persisted in our secondary multivariable regression analysis that examined decedents who received PC only by nonspecialists, excluding specialist consultation (eTable 5 in Supplement 1). In our multivariable regression analysis examining the association of the timing of specialist PC consultation (Table 4), compared with patients who received late specialist PC, patients receiving earlier specialist PC from 3 months up to 6 months before death were less likely to receive aggressive EOL care (OR, 0.59 [95% CI, 0.45-0.77]), more likely to receive supportive EOL care (OR, 1.52 [95% CI, 1.23-1.88]), and less likely to die in the hospital (OR, 0.66 [95% CI, 0.54-0.79]). These associations were also present for patients receiving earlier PC from 6 months up to 12 months before death and 12 or more months before death. Only patients who received specialist PC from 3 to 6 months before death were significantly less likely to receive late chemotherapy than patients receiving late specialist PC (OR, 0.46 [95% CI, 0.24-0.88]), while patients who did not receive a specialist PC consultation (including those who had nonspecialist PC only and those who had no PC at all) were at highest risk of receiving late chemotherapy (OR, 1.78 [95% CI, 1.30-2.42]).

**Table 4.** Multivariable Logistic Regression Analysis for Aggregate and Individual End-Of-Life Quality Indicators, Including Consultation by Specialist Palliative Care Physicians

Indicator	OR (95% CI)				
	Aggressive care	Supportive care	New ICU admission (last 30 d)	Chemotherapy use (last 14 d)	Death in hospital
Age group at death, y					
18-39	2.29 (1.47-3.58)	1.52 (0.91-2.54)	2.52 (1.22-5.22)	0.72 (0.22-2.38)	1.77 (1.18-2.67)
40-49	1.37 (1.04-1.82)	1.04 (0.80-1.36)	1.74 (1.06-2.83)	1.38 (0.82-2.33)	1.49 (1.19-1.88)
50-59	1 [Reference]	1 [Reference]	1 [Reference]	1 [Reference]	1 [Reference]
60-69	0.89 (0.73-1.07)	0.89 (0.75-1.05)	0.80 (0.55-1.16)	1.10 (0.78-1.56)	0.88 (0.75-1.02)
70-79	0.71 (0.59-0.87)	0.75 (0.64-0.89)	0.60 (0.40-0.88)	0.64 (0.44-0.94)	0.79 (0.68-0.92)
≥80	0.51 (0.41-0.64)	0.63 (0.53-0.75)	0.39 (0.24-0.62)	0.34 (0.21-0.55)	0.55 (0.47-0.65)
Oncologic survival from cancer diagnosis to death					
≥3 up to 6 mo	0.90 (0.68-1.20)	1.06 (0.84-1.34)	1.14 (0.63-2.06)	1.37 (0.81-2.31)	1.17 (0.94-1.46)
≥6 up to 12 mo	1 [Reference]	1 [Reference]	1 [Reference]	1 [Reference]	1 [Reference]
≥1 up to 5 y	0.72 (0.60-0.88)	1.36 (1.15-1.60)	0.87 (0.58-1.32)	0.90 (0.62-1.31)	0.86 (0.74-1.00)
≥5 y	0.68 (0.53-0.87)	1.24 (1.01-1.52)	1.20 (0.74-1.95)	0.75 (0.46-1.22)	0.83 (0.68-1.00)
Deyo-Charlson Comorbidity Index score^a^					
0 or Missing	1 [Reference]	1 [Reference]	1 [Reference]	1 [Reference]	1 [Reference]
≥1	0.89 (0.78-1.02)	0.99 (0.89-1.11)	0.91 (0.70-1.18)	0.89 (0.69-1.15)	0.95 (0.86-1.05)
Cancer stage					
I	1.37 (0.97-1.95)	0.95 (0.69-1.31)	1.01 (0.51-2.02)	0.71 (0.30-1.68)	1.02 (0.76-1.38)
II	0.91 (0.62-1.32)	0.91 (0.67-1.24)	1.24 (0.66-2.33)	1.44 (0.78-2.67)	0.89 (0.66-1.19)
III	1 [Reference]	1 [Reference]	1 [Reference]	1 [Reference]	1 [Reference]
IV	0.99 (0.80-1.22)	1.13 (0.95-1.36)	0.68 (0.43-1.08)	1.12 (0.76-1.67)	0.91 (0.77-1.08)
Missing	0.97 (0.81-1.15)	0.98 (0.85-1.13)	0.85 (0.60-1.19)	0.84 (0.59-1.18)	1.03 (0.90-1.17)
Rural status and income quintile					
Rural	1.63 (1.30-2.05)	0.87 (0.71-1.06)	0.55 (0.33-0.91)	1.16 (0.73-1.85)	1.61 (1.35-1.93)
Urban 1 (lowest)	1.42 (1.14-1.77)	0.62 (0.51-0.74)	1.03 (0.67-1.57)	1.41 (0.91-2.17)	1.19 (1.01-1.41)
Urban 2	1.08 (0.86-1.36)	0.69 (0.58-0.83)	0.85 (0.55-1.32)	1.04 (0.66-1.66)	1.00 (0.85-1.19)
Urban 3	1.12 (0.90-1.41)	0.75 (0.62-0.90)	1.01 (0.66-1.54)	1.46 (0.94-2.25)	1.15 (0.97-1.37)
Urban 4	1.03 (0.82-1.29)	0.84 (0.70-1.00)	0.90 (0.58-1.37)	1.25 (0.81-1.93)	1.07 (0.91-1.27)
Urban 5 (highest)	1 [Reference]	1 [Reference]	1 [Reference]	1 [Reference]	1 [Reference]
Timing of palliative care initiation prior to death					
No palliative care^b^	1.12 (0.96-1.31)	0.77 (0.68-0.88)	2.15 (1.55-2.99)	1.78 (1.30-2.42)	0.92 (0.81-1.04)
<3 mo	1 [Reference]	1 [Reference]	1 [Reference]	1 [Reference]	1 [Reference]
≥3 to 6 mo	0.59 (0.45-0.77)	1.52 (1.23-1.88)	0.73 (0.41-1.31)	0.46 (0.24-0.88)	0.66 (0.54-0.79)
≥6 to 12 mo	0.57 (0.42-0.77)	1.61 (1.27-2.04)	0.73 (0.38-1.41)	0.68 (0.36-1.28)	0.68 (0.56-0.84)
≥12 mo	0.74 (0.57-0.97)	1.37 (1.10-1.72)	0.90 (0.51-1.59)	0.70 (0.39-1.28)	0.73 (0.59-0.89)
Primary care clinician					
No	1.05 (0.89-1.23)	0.85 (0.74-0.97)	1.09 (0.79-1.50)	0.97 (0.71-1.33)	1.06 (0.94-1.20)
Yes	1 [Reference]	1 [Reference]	1 [Reference]	1 [Reference]	1 [Reference]
Year of death					
2006	1 [Reference]	1 [Reference]	1 [Reference]	1 [Reference]	1 [Reference]
2007	0.94 (0.66-1.32)	1.03 (0.78-1.35)	1.19 (0.58-2.45)	0.84 (0.47-1.50)	0.79 (0.61-1.02)
2008	1.03 (0.73-1.45)	0.97 (0.73-1.27)	1.23 (0.59-2.58)	0.82 (0.45-1.49)	1.15 (0.89-1.49)
2009	0.83 (0.58-1.19)	1.18 (0.89-1.56)	1.18 (0.56-2.47)	0.46 (0.23-0.92)	0.83 (0.63-1.07)
2010	0.96 (0.67-1.36)	1.23 (0.92-1.63)	1.07 (0.49-2.33)	0.58 (0.30-1.10)	0.71 (0.54-0.92)
2011	1.01 (0.71-1.43)	1.59 (1.19-2.13)	1.16 (0.54-2.50)	0.66 (0.35-1.25)	0.73 (0.56-0.96)
2012	1.04 (0.73-1.49)	1.62 (1.20-2.18)	1.68 (0.82-3.47)	0.49 (0.24-0.97)	0.70 (0.53-0.92)
2013	1.03 (0.72-1.47)	1.38 (1.03-1.84)	1.94 (0.95-3.98)	0.53 (0.27-1.04)	0.75 (0.58-0.99)
2014	1.04 (0.73-1.49)	1.37 (1.02-1.83)	1.68 (0.80-3.51)	0.53 (0.27-1.07)	0.73 (0.56-0.96)
2015	1.09 (0.76-1.55)	1.59 (1.19-2.13)	2.09 (1.02-4.30)	0.63 (0.33-1.24)	0.71 (0.54-0.93)
2016	1.14 (0.80-1.61)	1.57 (1.17-2.10)	1.13 (0.51-2.49)	0.75 (0.40-1.42)	0.61 (0.46-0.80)
2017	1.19 (0.84-1.68)	1.71 (1.28-2.28)	1.84 (0.89-3.81)	0.64 (0.33-1.23)	0.62 (0.47-0.82)
2018	1.29 (0.91-1.82)	1.58 (1.18-2.11)	2.20 (1.08-4.49)	0.86 (0.46-1.60)	0.73 (0.56-0.96)

Abbreviations: ICU, intensive care unit; OR, odds ratio

^a^ Scores may range from 0 to 17, with comorbidities present defined as a score of 1 or higher.

^b^ Patients who received no specialist palliative care included patients who received palliative care from nonspecialist clinicians only and patients who received no palliative care.

## Discussion

In this population-based cohort study of 8297 ovarian cancer decedents, we found that in comparison with patients who received earlier palliative care, patients for whom palliative care was initiated in the final 3 months of life were twice as likely to receive aggressive EOL care. These findings remained significant even when examining PC exclusively delivered or initiated by nonspecialist PC clinicians. There was no difference in the likelihood of dying in the hospital or receiving aggressive EOL care between patients who received late PC and patients who received no PC. Early specialist consultation from 3 months up to 6 months before death was also associated with decreased likelihood of late chemotherapy compared with later or no specialist consultation.

Although we uniquely examined late vs early PC using definitions of less than 3 months prior to death vs 3 or more months prior to death that align with contemporary recommendations,^1,23,27^ our findings are supported by previous studies.^13,28,29,30,31^ Two population-based studies in gynecologic oncology examined the association of PC at the EOL (≤30 days before death) vs earlier PC.^28,29^ A study by Vestergaard et al^29^ of over 4500 Danish gynecologic cancer decedents reported that earlier initiation of hospital-based specialist PC was associated with less oncologic treatment in the final 14 days, fewer ED visits or admissions to the hospital or ICU at the EOL, less death in acute care, and more hospice admission. The study by Paulsen et al^28^ of 163 Norwegian decedents reported that earlier initiation of specialist PC was associated with more documented goals of care conversations and lower ICU admission rates, but in contrast to our findings, no difference in late chemotherapy.

Our findings suggest that the timing of PC initiation matters. As late PC initiation may be less effective in improving the quality of EOL care, and may not be associated with key EOL outcomes compared with no PC, it may represent a suboptimal use of finite PC resources. One explanation is that time is required to build relationships with patients that facilitate challenging discussions about EOL care goals and appropriate care de-escalation.^3^ Establishing community resources and supports for symptom management and death at home or in hospice also requires time and planning. Earlier initiation of PC may thus reduce the need for EOL ED visits or admissions.^32,33^ In our cohort, one-third of patients initiated PC late, and prior studies suggest that 30% to 80% of gynecologic cancer decedents initiate PC only in the final month of life.^13,30,34^ A substantial proportion of oncology patients may be initiating PC too late to achieve high-quality EOL care.

### Strengths and Limitations

A strength of the present study is the examination of PC provision by both specialist and nonspecialist clinicians, as most studies have examined only specialist referral.^28,29,30,31,34^ Even with the exclusion of patients who received specialist consultation, early PC remained associated with less-aggressive EOL care. This supports contemporary models of PC delivery that recommend early initiation of interdisciplinary primary PC by the treating oncology team, reserving the finite resource of specialist care for refractory or complex cases.^1,35^ This finding also underscores the importance of broadly integrating PC training into medical education, including surgical, radiation, and medical oncology programs, particularly in the face of a shortage of specialty-trained palliative physicians.^35^ Our finding that only patients who received early specialist PC consultation were less likely to receive late chemotherapy is hypothesis generating; it may indicate that patients deemed less appropriate by their oncologist for chemotherapy are referred to PC earlier, or that earlier PC by a specialist outside the treating oncologic team may influence decision-making around the role of anticancer treatment at EOL.

Limitations of the present study include the use of retrospective administrative data that do not capture private-payer services and may not capture all relevant EOL quality indicators. Hospice care in Ontario includes services and supports provided across a variety of settings^36^ and was captured in administrative codes for home care provided in hospice or PC units as well as services in long-term and continuing care facilities. Thus, we could not quantify hospice enrollment. As our administrative databases do not capture timing of cancer recurrence or treatment intent for all modalities, we were unable to examine timing of PC relative to recurrences. There were no cancer stage data available for 52.3% of our cohort, in part because these data were not captured in the Ontario Cancer Registry in 2006 and most of 2007. To address this source of bias, we characterized the cohort with missing cancer stage data and found no association between cancer stage and EOL outcomes in our secondary regression analysis of patients with known stage data. Administrative data limited the analysis of the association of PC timing with patient-reported outcomes, and the concordance of EOL care with patient and caregiver preferences. By separately examining the cohort of patients who did not receive any PC, which may indicate a care preference, we attempted to mitigate the impact of this bias. Although our data are population-based, we were unable to capture race or ethnicity data, and our findings may not be generalizable to other health care jurisdictions.

## Conclusions

Findings of this cohort study indicated that early initiation of PC earlier than 3 months before death was associated with improved quality and decreased intensity of care at the EOL among ovarian cancer decedents. With increasing access to clinical trials and rapid uptake of therapeutic options, including targeted and immune therapies that prolong survival in patients with advanced cancer, future research is warranted to improve prognostication and prospective identification of candidates for PC, and strategies are needed to address barriers to early PC, including patient factors, oncologist beliefs and practice patterns, and system or PC resource constraints.^37,38^

## Notes

Supplement 1. **eTable 1.** Characteristics of ovarian cancer decedents with missing stage data **eTable 2.** Timing of palliative care initiation and receipt of specialist palliative care consultation by cancer stage category (early stage I-II, advanced stage III-IV, and missing stage data) **eTable 3.** Multivariable logistic regression for aggregate and individual end-of-life quality indicators including all palliative care in ovarian cancer decedents with missing cancer stage data **eTable 4.** Multivariable logistic regression for aggregate and individual end-of-life quality indicators including all palliative care in ovarian cancer decedents with known cancer stage **eTable 5.** Multivariable logistic regression for aggregate and individual end-of-life quality indicators including non-specialist palliative care only

Supplement 2.
Data Sharing Statement
