# Supplementary material for: Timing of Palliative Care, End-of-Life Quality Indicators, and Health Resource Utilization
Source: JAMA Netw Open. 2024 Oct 28;7(10):e2440977. doi: 10.1001/jamanetworkopen.2024.40977 (PMC11519754; doi:10.1001/jamanetworkopen.2024.40977)
Supplement: Supplement 1. — eTable 1. Characteristics of ovarian cancer decedents with missing stage data eTable 2. Timing of palliative care initiation and receipt of specialist palliative care consultation by cancer stage category (early stage I-II, advanced stage III-IV, and missing stage data) eTable 3. Multivariable logistic regression for aggregate and individual end-of-life quality indicators including all palliative care in ovarian cancer decedents with missing cancer stage data eTable 4. Multivariable logistic regression for aggregate and individual end-of-life quality indicators including all palliative care in ovarian cancer decedents with known cancer stage eTable 5. Multivariable logistic regression for aggregate and individual end-of-life quality indicators including non-specialist palliative care only [file jamanetwopen-e2440977-s001.pdf]

Supplementary Online Content

Mah J, Carter Ramirez DM, Schnarr K, Eiriksson LR, Gayowsky A, Seow H. Timing of palliative care, end-of-life quality indicators, and health resource utilization. *JAMA Netw Open*. 2024;7(10):e2440977. doi:10.1001/jamanetworkopen.2024.40977

eTable 1. Characteristics of ovarian cancer decedents with missing stage data
eTable 2. Timing of palliative care initiation and receipt of specialist palliative care consultation by cancer stage category (early stage I-II, advanced stage III-IV, and missing stage data)
eTable 3. Multivariable logistic regression for aggregate and individual end-of-life quality indicators including all palliative care in ovarian cancer decedents with missing cancer stage data
eTable 4. Multivariable logistic regression for aggregate and individual end-of-life quality indicators including all palliative care in ovarian cancer decedents with known cancer stage
eTable 5. Multivariable logistic regression for aggregate and individual end-of-life quality indicators including non-specialist palliative care only

This supplementary material has been provided by the authors to give readers additional information about their work.

© 2024 Mah J et al. *JAMA Network Open*.

eTable 1. Characteristics of ovarian cancer decedents with missing stage data

Variable	Category	n= 4,339	%
**Age at death (years)**	18-39	51	1.2
	40-49	232	5.4
	50-59	610	14.1
	60-69	971	22.4
	70-79	1,163	26.8
	80+	1,312	30.2
**Length of survival from gynecologic cancer diagnosis**	< 30 days	733	12.8
	≥ 1-3 months	843	12.6
	≥ 3-6 months	568	6.5
	≥ 6-12 months	864	9.0
	≥ 12 months - 5 years	3,968	37.2
	≥ 5 years	1,321	21.9
**Timing of palliative care initiation prior to death**	No palliative care	327	6.0
	< 30 days	1,304	21.0
	≥ 1-3 months	1,363	19.6
	≥ 3-6 months	947	11.9
	≥ 6-12 months	1,175	12.6
	≥ 12 months	3,181	28.9
**Score on the Deyo-Charlson comorbidity index (from -36 months to -1 months before death)**	0	1,950	22.3
	1+	3,809	47.1
	Unknown/Missing Data	2,538	30.7
**Patient has a primary care provider in CAPE as of diagnosis date**	Yes	5,939	60.6
	No	2,358	39.4
**Rural status and income quintile**	Rural	1,131	13.6
	Urban Income Quintile 1 (lowest)	1,484	18.0
	Urban Income Quintile 2	1,459	17.9
	Urban Income Quintile 3	1,357	16.6
	Urban Income Quintile 4	1,415	16.8
	Urban Income Quintile 5 (highest)	1,433	16.8
	Unknown/Missing Data	18	0.3

© 2024 Mah J et al. *JAMA Network Open*.

eTable 2. Timing of palliative care initiation and receipt of specialist palliative care consultation by cancer stage category (early stage I-II, advanced stage III-IV, and missing stage data)

		Stages I-II	Stages III-IV	Missing Stage	Total
		**N=463 n (%)**	**N=3,495 n (%)**	**N=4,339 n (%)**	**N=8,297 n (%)**
**Timing of palliative care initiation prior to death**	No palliative care	9 (1.9)	57 (1.6)	261 (6.0)	327 (3.9)
	< 30 days	37 (8.0)	358 (10.2)	909 (21.0)	1,304 (15.7)
	≥ 1-3 months	57 (12.3)	457 (13.1)	849 (19.6)	1,363 (16.4)
	≥ 3-6 months	58 (12.5)	371 (10.6)	518 (11.9)	947 (11.4)
	≥ 6-12 months	76 (16.4)	552 (15.8)	547 (12.6)	1,175 (14.2)
	≥ 12 months	226 (48.8)	1,700 (48.6)	1,255 (28.9)	3,181 (38.3)
**Specialist Palliative Care Consultation**	No specialist consult	189 (40.8)	1,368 (39.1)	2,115 (48.7)	3,672 (44.3)
	Specialist consult	265 (57.2)	2,070 (59.2)	1,963 (45.2)	4,298 (51.8)
	No palliative care	9 (1.9)	57 (1.6)	261 (6.0)	327 (3.9)

© 2024 Mah J et al. *JAMA Network Open*.

eTable 3. Multivariable logistic regression for aggregate and individual end-of-life quality indicators including all palliative care in ovarian cancer decedents with missing cancer stage data

		**Aggressive Care** OR (95% CI)	**Supportive Care** OR (95% CI)	**New ICU Admission (last 30 days)** OR (95% CI)	**Chemotherapy Use (last 14 days)** OR (95% CI)	**Death in Hospital** OR (95% CI)
**Age at Death (years)**	18-39	2.40 (1.22-4.72)	2.27 (0.96-5.34)	1.24 (0.28-5.57)	0.42 (0.05-3.28)	1.53 (0.81-2.89)
	40-49	1.05 (0.69-1.59)	1.18 (0.80-1.74)	1.41 (0.68-2.96)	1.15 (0.55-2.40)	1.36 (0.97-1.89)
	50-59 (REF)	1.00	1.00	1.00	1.00	1.00
	60-69	0.79 (0.59-1.06)	0.92 (0.71-1.19)	0.67 (0.37-1.20)	0.90 (0.54-1.51)	0.80 (0.64-1.00)
	70-79	0.72 (0.54-0.96)	0.73 (0.56-0.93)	0.75 (0.42-1.31)	0.70 (0.41-1.19)	0.76 (0.61-0.95)
	80+	0.43 (0.31-0.60)	0.54 (0.42-0.70)	0.36 (0.18-0.70)	0.20 (0.10-0.44)	0.52 (0.41-0.65)
**Oncologic Survival from Cancer Diagnosis to Death**	≥ 3-6 months	0.75 (0.47-1.19)	1.13 (0.76-1.68)	0.51 (0.18-1.46)	1.79 (0.73-4.41)	1.12 (0.78-1.59)
	≥ 6-12 months (REF)	1.00	1.00	1.00	1.00	1.00
	≥ 1-5 years	0.71 (0.50-0.99)	1.17 (0.87-1.56)	0.66 (0.33-1.31)	1.09 (0.56-2.14)	0.79 (0.61-1.02)
	≥ 5 years	0.59 (0.40-0.88)	1.26 (0.90-1.77)	0.86 (0.41-1.83)	0.69 (0.31-1.52)	0.71 (0.53-0.96)
**Deyo-Charlson Score**	0 or Missing (REF)	1.00	1.00	1.00	1.00	1.00
	≥ 1	0.99 (0.82-1.20)	0.91 (0.78-1.07)	1.07 (0.72-1.59)	1.03 (0.71-1.50)	0.95 (0.82-1.09)
**Rural Status and Income Quintile**	Rural	2.06 (1.47-2.90)	0.64 (0.48-0.87)	0.68 (0.32-1.45)	1.39 (0.70-2.73)	1.86 (1.44-2.42)
	Urban 1 (lowest)	1.45 (1.05-2.02)	0.55 (0.42-0.72)	0.83 (0.42-1.62)	1.52 (0.82-2.79)	1.14 (0.90-1.46)
	Urban 2	1.13 (0.80-1.58)	0.63 (0.48-0.83)	0.84 (0.44-1.62)	0.86 (0.43-1.72)	1.05 (0.82-1.34)
	Urban 3	1.41 (1.01-1.96)	0.66 (0.50-0.87)	1.24 (0.68-2.27)	1.56 (0.84-2.88)	1.27 (0.99-1.62)
	Urban 4	1.15 (0.82-1.62)	0.89 (0.68-1.17)	0.93 (0.49-1.78)	0.74 (0.36-1.51)	1.14 (0.89-1.45)
	Urban 5 (highest) (REF)	1.00	1.00	1.00	1.00	1.00
**Timing of Palliative Care Initiation**	No palliative care	1.01 (0.65-1.57)	NA	5.45 (2.89-10.28)	2.74 (1.39-5.40)	0.86 (0.60-1.25)
	< 3 months (REF)	1.00	1.00	1.00	1.00	1.00
	≥ 3-6 months	0.52 (0.38-0.72)	2.23 (1.71-2.91)	0.62 (0.30-1.29)	0.60 (0.31-1.15)	0.50 (0.39-0.64)

© 2024 Mah J et al. *JAMA Network Open*.

**Prior to Death**	≥ 6-12 months	0.48 (0.34-0.66)	2.20 (1.69-2.88)	0.52 (0.25-1.08)	0.81 (0.44-1.50)	0.61 (0.48-0.77)
	≥ 12 months	0.53 (0.40-0.69)	2.06 (1.65-2.58)	0.66 (0.37-1.16)	0.67 (0.39-1.15)	0.59 (0.48-0.72)
**Primary Care Provider**	No	1.00 (0.79-1.25)	0.89 (0.73-1.08)	1.17 (0.74-1.86)	1.39 (0.90-2.15)	1.13 (0.95-1.35)
	Yes (REF)	1.00	1.00	1.00	1.00	1.00
**Year of Death**	2006 (REF)	1.00	1.00	1.00	1.00	1.00
	2007	0.93 (0.65-1.32)	1.04 (0.77-1.40)	1.24 (0.59-2.60)	0.77 (0.42-1.43)	0.77 (0.59-1.01)
	2008	0.93 (0.63-1.37)	0.92 (0.67-1.27)	1.31 (0.58-2.99)	0.88 (0.44-1.76)	1.22 (0.92-1.62)
	2009	0.86 (0.57-1.31)	1.06 (0.75-1.49)	1.38 (0.57-3.34)	0.72 (0.32-1.62)	0.96 (0.71-1.30)
	2010	1.05 (0.67-1.67)	0.95 (0.66-1.37)	1.82 (0.72-4.61)	0.22 (0.05-0.97)	0.76 (0.54-1.07)
	2011	1.04 (0.63-1.71)	1.16 (0.77-1.74)	1.39 (0.47-4.08)	0.91 (0.35-2.36)	1.03 (0.72-1.48)
	2012	1.30 (0.78-2.17)	1.15 (0.74-1.78)	2.20 (0.82-5.93)	1.27 (0.50-3.17)	0.99 (0.67-1.44)
	2013	1.38 (0.82-2.31)	0.98 (0.64-1.50)	2.62 (0.97-7.09)	0.53 (0.15-1.85)	0.63 (0.42-0.96)
	2014	1.41 (0.85-2.36)	1.01 (0.66-1.54)	2.66 (0.99-7.19)	0.49 (0.14-1.72)	0.86 (0.58-1.27)
	2015	1.42 (0.88-2.30)	1.27 (0.84-1.91)	2.76 (1.06-7.21)	1.04 (0.42-2.59)	0.83 (0.57-1.21)
	2016	1.23 (0.76-1.98)	1.02 (0.69-1.51)	1.56 (0.52-4.69)	0.83 (0.32-2.17)	0.68 (0.47-0.98)
	2017	1.40 (0.88-2.25)	1.66 (1.10-2.51)	2.85 (1.09-7.45)	1.15 (0.48-2.74)	0.67 (0.46-0.97)
	2018	1.40 (0.88-2.20)	1.30 (0.87-1.93)	1.20 (0.37-3.92)	1.13 (0.49-2.62)	0.84 (0.58-1.20)

© 2024 Mah J et al. *JAMA Network Open*.

eTable 4. Multivariable logistic regression for aggregate and individual end-of-life quality indicators including all palliative care in ovarian cancer decedents with known cancer stage

		Aggressive Care OR (95% CI)	Supportive Care OR (95% CI)	New ICU Admission (last 30 days) OR (95% CI)	Chemotherapy Use (last 14 days) OR (95% CI)	Death in Hospital OR (95% CI)
**Age at Death (years)**	18-39	2.13 (1.17-3.87)	1.37 (0.69-2.72)	3.04 (1.27-7.27)	0.90 (0.20-3.97)	1.89 (1.10-3.24)
	40-49	1.63 (1.11-2.40)	1.09 (0.73-1.63)	1.69 (0.86-3.34)	1.35 (0.65-2.82)	1.56 (1.13-2.16)
	50-59 (REF)	1.00	1.00	1.00	1.00	1.00
	60-69	0.98 (0.76-1.26)	0.85 (0.67-1.07)	0.90 (0.55-1.46)	1.29 (0.80-2.08)	0.95 (0.77-1.16)
	70-79	0.73 (0.56-0.95)	0.71 (0.56-0.89)	0.52 (0.30-0.90)	0.66 (0.38-1.13)	0.84 (0.68-1.03)
	80+	0.62 (0.46-0.84)	0.71 (0.55-0.91)	0.41 (0.21-0.80)	0.54 (0.29-1.01)	0.59 (0.47-0.75)
**Oncologic Survival from Cancer Diagnosis to Death**	≥ 3-6 months	1.00 (0.64-1.54)	1.16 (0.80-1.70)	1.70 (0.72-4.04)	1.10 (0.51-2.41)	1.28 (0.90-1.82)
	≥ 6-12 months (REF)	1.00	1.00	1.00	1.00	1.00
	≥ 1-5 years	0.78 (0.57-1.06)	1.43 (1.09-1.87)	1.30 (0.67-2.52)	0.71 (0.39-1.29)	0.88 (0.68-1.13)
	≥ 5 years	0.82 (0.54-1.24)	1.23 (0.85-1.76)	1.35 (0.58-3.13)	0.64 (0.28-1.47)	0.80 (0.57-1.11)
**Deyo-Charlson Score**	0 or Missing (REF)	1.00	1.00	1.00	1.00	1.00
	≥ 1	0.85 (0.71-1.02)	0.97 (0.83-1.13)	0.96 (0.67-1.39)	0.77 (0.54-1.10)	0.97 (0.84-1.12)
**Cancer Stage**	I	1.33 (0.93-1.89)	1.00 (0.72-1.39)	0.88 (0.43-1.80)	0.75 (0.32-1.78)	1.02 (0.76-1.38)
	II	0.94 (0.65-1.38)	0.88 (0.64-1.19)	1.37 (0.72-2.60)	1.57 (0.85-2.90)	0.90 (0.67-1.21)
	III (REF)	1.00	1.00	1.00	1.00	1.00
	IV	1.03 (0.83-1.27)	1.11 (0.92-1.33)	0.70 (0.44-1.12)	1.16 (0.78-1.73)	0.92 (0.78-1.09)
	Rural	1.60 (1.17-2.17)	0.91 (0.68-1.21)	0.63 (0.32-1.25)	1.36 (0.72-2.60)	1.50 (1.17-1.92)

© 2024 Mah J et al. *JAMA Network Open*.

**Rural Status and Income Quintile**	Urban 1 (lowest)	1.37 (1.02-1.84)	0.64 (0.49-0.83)	1.18 (0.67-2.07)	1.17 (0.63-2.18)	1.23 (0.97-1.55)
	Urban 2	1.10 (0.81-1.50)	0.67 (0.51-0.87)	0.88 (0.48-1.61)	1.19 (0.63-2.22)	1.00 (0.79-1.27)
	Urban 3	0.89 (0.65-1.22)	0.82 (0.63-1.07)	0.75 (0.40-1.39)	1.31 (0.71-2.45)	1.06 (0.83-1.35)
	Urban 4	0.97 (0.72-1.32)	0.71 (0.55-0.92)	0.91 (0.51-1.63)	1.80 (1.01-3.20)	1.06 (0.84-1.35)
	Urban 5 (highest) (REF)	1.00	1.00	1.00	1.00	1.00
**Timing of Palliative Care Initiation Prior to Death**	No palliative care	1.90 (0.85-4.26)	NA	4.12 (1.48-11.48)	3.04 (0.91-10.10)	1.01 (0.46-2.23)
	< 3 months (REF)	1.00	1.00	1.00	1.00	1.00
	≥ 3-6 months	0.43 (0.30-0.62)	1.73 (1.28-2.34)	0.33 (0.16-0.68)	1.06 (0.54-2.07)	0.60 (0.45-0.80)
	≥ 6-12 months	0.55 (0.40-0.76)	1.90 (1.42-2.54)	0.39 (0.20-0.75)	0.87 (0.44-1.71)	0.66 (0.51-0.87)
	≥ 12 months	0.53 (0.39-0.71)	1.93 (1.48-2.50)	0.33 (0.19-0.56)	1.12 (0.60-2.07)	0.70 (0.55-0.90)
**Primary Care Provider**	No	1.12 (0.89-1.42)	0.80 (0.65-0.98)	0.93 (0.57-1.51)	0.63 (0.37-1.09)	0.98 (0.81-1.19)
	Yes (REF)	1.00	1.00	1.00	1.00	1.00
**Year of Death**	2007/2008 (REF)	1.00	1.00	1.00	1.00	1.00
	2009	0.72 (0.41-1.29)	1.08 (0.66-1.75)	1.51 (0.41-5.50)	0.34 (0.11-1.03)	0.68 (0.43-1.06)
	2010	0.89 (0.52-1.52)	1.08 (0.68-1.70)	1.06 (0.28-4.00)	0.83 (0.36-1.94)	0.69 (0.45-1.04)
	2011	1.01 (0.60-1.69)	1.39 (0.89-2.18)	1.62 (0.47-5.53)	0.60 (0.25-1.42)	0.63 (0.42-0.95)
	2012	1.00 (0.59-1.69)	1.36 (0.86-2.14)	2.59 (0.79-8.51)	0.33 (0.13-0.88)	0.62 (0.41-0.93)
	2013	0.97 (0.58-1.62)	1.13 (0.72-1.75)	2.82 (0.88-9.12)	0.48 (0.20-1.17)	0.84 (0.56-1.26)
	2014	0.94 (0.56-1.59)	1.14 (0.73-1.78)	2.24 (0.67-7.48)	0.48 (0.20-1.20)	0.72 (0.48-1.08)
	2015	0.92 (0.55-1.56)	1.41 (0.89-2.22)	2.69 (0.82-8.87)	0.39 (0.15-0.99)	0.67 (0.44-1.01)
	2016	1.08 (0.64-1.82)	1.48 (0.94-2.34)	1.54 (0.43-5.50)	0.62 (0.26-1.49)	0.60 (0.40-0.91)
	2017	1.09 (0.65-1.82)	1.30 (0.83-2.04)	2.51 (0.75-8.34)	0.39 (0.16-0.98)	0.62 (0.41-0.94)
	2018	1.21 (0.72-2.05)	1.35 (0.85-2.14)	4.14 (1.28-13.40)	0.58 (0.24-1.42)	0.73 (0.48-1.10)

© 2024 Mah J et al. *JAMA Network Open*.

eTable 5. Multivariable logistic regression for aggregate and individual end-of-life quality indicators including non-specialist palliative care only

		Aggressive Care OR (95% CI)	Supportive Care OR (95% CI)	New ICU Admission (last 30 days) OR (95% CI)	Chemotherapy Use (last 14 days) OR (95% CI)	Death in Hospital OR (95% CI)
Age at Death (years)	18-39	2.26 (1.45-3.53)	1.63 (0.96-2.78)	2.43 (1.17-5.06)	0.65 (0.20-2.16)	1.75 (1.16-2.64)
	40-49	1.33 (1.00-1.76)	1.14 (0.86-1.51)	1.56 (0.95-2.57)	1.25 (0.75-2.10)	1.47 (1.17-1.86)
	50-59 (REF)	1.00	1.00	1.00	1.00	1.00
	60-69	0.90 (0.74-1.09)	0.88 (0.74-1.04)	0.82 (0.57-1.19)	1.12 (0.79-1.59)	0.88 (0.76-1.03)
	70-79	0.73 (0.60-0.89)	0.72 (0.60-0.85)	0.65 (0.44-0.96)	0.69 (0.48-1.00)	0.80 (0.69-0.93)
	80+	0.52 (0.42-0.65)	0.62 (0.52-0.74)	0.39 (0.25-0.63)	0.36 (0.22-0.58)	0.56 (0.47-0.66)
Oncologic Survival from Cancer Diagnosis to Death	≥ 3-6 months	0.87 (0.63-1.19)	1.16 (0.88-1.53)	1.01 (0.53-1.92)	1.42 (0.79-2.53)	1.19 (0.93-1.53)
	≥ 6-12 months (REF)	1.00	1.00	1.00	1.00	1.00
	≥ 1-5 years	0.73 (0.58-0.91)	1.34 (1.10-1.63)	0.92 (0.58-1.47)	0.90 (0.59-1.38)	0.84 (0.70-1.00)
	≥ 5 years	0.66 (0.50-0.86)	1.32 (1.04-1.67)	1.15 (0.68-1.97)	0.70 (0.41-1.20)	0.78 (0.63-0.97)
Deyo-Charlson Score	0 or Missing (REF)	1.00	1.00	1.00	1.00	1.00
	≥ 1	0.91 (0.80-1.04)	0.94 (0.84-1.05)	0.98 (0.75-1.28)	0.90 (0.70-1.16)	0.96 (0.87-1.06)
Cancer Stage	I	1.34 (0.95-1.90)	1.00 (0.72-1.38)	0.95 (0.47-1.91)	0.73 (0.31-1.71)	1.01 (0.75-1.36)
	II	0.94 (0.64-1.37)	0.87 (0.64-1.19)	1.36 (0.72-2.55)	1.57 (0.85-2.89)	0.91 (0.68-1.22)
	III (REF)	1.00	1.00	1.00	1.00	1.00
	IV	1.01 (0.82-1.25)	1.10 (0.91-1.32)	0.73 (0.46-1.15)	1.15 (0.78-1.71)	0.92 (0.78-1.09)
	Missing	0.99 (0.83-1.17)	0.97 (0.84-1.12)	0.87 (0.62-1.22)	0.89 (0.63-1.25)	1.04 (0.92-1.19)
Rural Status and Income Quintile	Rural	1.79 (1.43-2.25)	0.78 (0.63-0.95)	0.65 (0.39-1.08)	1.36 (0.85-2.16)	1.66 (1.39-1.98)
	Urban 1 (lowest)	1.42 (1.14-1.77)	0.60 (0.50-0.72)	1.03 (0.67-1.57)	1.35 (0.88-2.08)	1.19 (1.01-1.41)
	Urban 2	1.12 (0.89-1.40)	0.66 (0.54-0.79)	0.85 (0.55-1.32)	1.03 (0.65-1.63)	1.02 (0.86-1.21)
	Urban 3	1.13 (0.90-1.42)	0.74 (0.61-0.89)	0.97 (0.63-1.49)	1.41 (0.92-2.18)	1.16 (0.98-1.37)
	Urban 4	1.05 (0.84-1.32)	0.79 (0.66-0.96)	0.92 (0.60-1.41)	1.25 (0.81-1.93)	1.10 (0.93-1.30)

© 2024 Mah J et al. *JAMA Network Open*.

	Urban 5 (highest) (REF)	1.00	1.00	1.00	1.00	1.00
**Timing of Palliative Care Initiation Prior to Death**	No palliative care	1.18 (0.81-1.71)	NA	5.00 (2.99-8.35)	2.46 (1.39-4.36)	0.90 (0.65-1.24)
	< 3 months (REF)	1.00	1.00	1.00	1.00	1.00
	≥ 3-6 months	0.48 (0.38-0.61)	2.03 (1.66-2.47)	0.46 (0.27-0.76)	0.74 (0.47-1.16)	0.54 (0.45-0.65)
	≥ 6-12 months	0.52 (0.41-0.65)	2.10 (1.73-2.54)	0.44 (0.27-0.72)	0.81 (0.52-1.25)	0.62 (0.52-0.74)
	≥ 12 months	0.53 (0.43-0.64)	2.00 (1.69-2.36)	0.47 (0.32-0.70)	0.79 (0.53-1.17)	0.64 (0.55-0.74)
**Primary Care Provider**	No	1.03 (0.88-1.21)	0.86 (0.75-0.99)	1.07 (0.78-1.48)	0.97 (0.71-1.32)	1.06 (0.93-1.20)
	Yes (REF)	1.00	1.00	1.00	1.00	1.00
**Year of Death**	2006 (REF)	1.00	1.00	1.00	1.00	1.00
	2007	0.94 (0.66-1.32)	1.03 (0.76-1.38)	1.18 (0.56-2.46)	0.85 (0.47-1.52)	0.79 (0.61-1.02)
	2008	1.02 (0.72-1.44)	0.88 (0.66-1.18)	1.34 (0.63-2.86)	0.84 (0.46-1.53)	1.15 (0.89-1.49)
	2009	0.88 (0.61-1.26)	0.98 (0.73-1.32)	1.53 (0.72-3.26)	0.51 (0.26-1.03)	0.85 (0.66-1.11)
	2010	1.07 (0.75-1.54)	0.93 (0.69-1.26)	1.56 (0.71-3.44)	0.65 (0.34-1.24)	0.75 (0.58-0.99)
	2011	1.17 (0.82-1.67)	1.19 (0.88-1.62)	1.66 (0.76-3.66)	0.72 (0.38-1.38)	0.81 (0.62-1.06)
	2012	1.25 (0.86-1.80)	1.17 (0.85-1.60)	2.65 (1.25-5.61)	0.55 (0.27-1.10)	0.78 (0.59-1.03)
	2013	1.23 (0.85-1.76)	0.99 (0.73-1.34)	2.90 (1.38-6.10)	0.55 (0.28-1.10)	0.85 (0.64-1.12)
	2014	1.23 (0.85-1.77)	1.01 (0.74-1.36)	2.44 (1.14-5.25)	0.52 (0.26-1.06)	0.82 (0.62-1.08)
	2015	1.23 (0.86-1.76)	1.24 (0.91-1.69)	2.78 (1.32-5.88)	0.57 (0.29-1.13)	0.78 (0.59-1.02)
	2016	1.31 (0.92-1.87)	1.17 (0.86-1.59)	1.60 (0.70-3.62)	0.73 (0.38-1.39)	0.68 (0.51-0.89)
	2017	1.38 (0.97-1.96)	1.27 (0.94-1.72)	2.66 (1.25-5.67)	0.60 (0.31-1.18)	0.69 (0.53-0.91)
	2018	1.49 (1.05-2.12)	1.20 (0.89-1.63)	2.96 (1.41-6.21)	0.79 (0.42-1.49)	0.82 (0.62-1.08)

Patients who received "No palliative care" in this analysis received no palliative care at all from any provider type

© 2024 Mah J et al. *JAMA Network Open*.
