# Supplementary material for: Timing of Palliative Care, End-of-Life Quality Indicators, and Health Resource Utilization
Source: JAMA Netw Open. 2024 Oct 28;7(10):e2440977. doi: 10.1001/jamanetworkopen.2024.40977 (PMC11519754; doi:10.1001/jamanetworkopen.2024.40977)
Supplement: Supplement 2. — Data Sharing Statement [file jamanetwopen-e2440977-s002.pdf]

Data Sharing Statement

Data
Data available: No

Additional Information
Explanation for why data not available: ICES Data is tightly regulated and cannot be disseminated in disaggregate fashion
